# Machine Learning-Based Modeling of Ovarian Response and the Quantitative Evaluation of Comprehensive Impact Features

**DOI:** 10.3390/diagnostics12020492

**Published:** 2022-02-14

**Authors:** Liu Liu, Fujin Shen, Hua Liang, Zhe Yang, Jing Yang, Jiao Chen

**Affiliations:** 1 Department of Obstetrics and Gynecology, Renmin Hospital, Wuhan University, Wuhan 430072, China; drliuliu@whu.edu.cn (L.L.); shenfj_rmh@outlook.com (F.S.); lianghua027@outlook.com (H.L.); 2 Reproductive Medicine Center, Renmin Hospital, Wuhan University, Wuhan 430072, China; 2016302180220@whu.edu.cn

**Keywords:** machine learning, controlled ovarian stimulation, number of oocytes retrieved, dosage of Gn, clinical decision support

## Abstract

Appropriate ovarian responses to the controlled ovarian stimulation strategy is the premise for a good outcome of the in vitro fertilization cycle. With the booming of artificial intelligence, machine learning is becoming a popular and promising approach for tailoring a controlled ovarian stimulation strategy. Nowadays, most machine learning-based tailoring strategies aim to generally classify the controlled ovarian stimulation outcome, lacking the capacity to precisely predict the outcome and evaluate the impact features. Based on a clinical cohort composed of 1365 women and two machine learning methods of artificial neural network and supporting vector regression, a regression prediction model of the number of oocytes retrieved is trained, validated, and selected. Given the proposed model, an index called the normalized mean impact value is defined and calculated to reflect the importance of each impact feature. The proposed models can estimate the number of oocytes retrieved with high precision, with the regression coefficient being 0.882% and 89.84% of the instances having the prediction number ≤ 5. Among the impact features, the antral follicle count has the highest importance, followed by the E_2_ level on the human chorionic gonadotropin day, the age, and the Anti-Müllerian hormone, with their normalized mean impact value > 0.3. Based on the proposed model, the prognostic results for ovarian response can be predicted, which enables scientific clinical decision support for the customized controlled ovarian stimulation strategies for women, and eventually helps yield better in vitro fertilization outcomes.

## 1. Introduction

Since the first successful in vitro fertilization–embryo transfer (IVF-ET) in 1978, human-assisted reproductive technology (ART) has made rapid progress in the past decades and helped many infertile couples obtain offspring. The success of IVF is not only related to the laboratory culture conditions and the personnel operation [1,2] but is also closely related to the quality and quantity of oocytes. The ovarian response to controlled ovarian stimulation (COS) is an essential factor, as the retrieving of multiple oocytes stimulated by gonadotropin (Gn) is the fundamental operation for the high-quality embryo’s formation, selection, and transfer in a successful ART process.

Existing studies have demonstrated that the number of oocytes retrieved can significantly affect the probability of obtaining a live birth rate (LBR) with a fresh embryo transfer [3,4,5,6]. Studies also revealed that the retrieval of 15–18 oocytes could yield the optimal IVF outcomes [7]; therefore, tailoring the COS strategy for a target oocyte number is meaningful, which can be accomplished by customizing treatment strategies, such as the ovarian stimulation regimens, types of drugs, and the dosages. For example, the full potential of the ovary is expressed only when a large amount of Gn is applied, but this condition normally does not occur to avoid life-threatening complications, such as the severe ovarian hyperstimulation syndrome (OHSS) [7,8].

The ovarian response to COS is a quantitative reflection of the reserve function of the ovary. Poor ovarian response leads to a high risk of treatment cycles being canceled or a lack of high-quality embryos for transfer. Studies have demonstrated that the impact features, e.g., a women’s clinical information (age, body mass index (BMI), infertility cause, and infertility duration), basal endocrine level (Anti-Müllerian hormone (AMH), basal follicle stimulating hormone (bFSH)), and ultrasound-related index (antral follicle count (AFC)), are closely related to the extent of ovarian response to COS [9,10,11,12,13,14,15]. The features, such as age, AMH, bFSH, and AFC, etc., are currently recognized as high-impact features related to ovarian reserve function [10,16,17,18]. As the impact features have their characteristics and interact with each other, it is necessary to formulate a comprehensive and accurate relationship between them and the COS outcome, and this brings about new challenges to clinical practice. In recent years, there are booming works on building machine learning-based clinical decision models for the IVF [19,20,21,22], considering the relevant prognostic features. The machine learning algorithms, such as the artificial neural network (ANN), supporting vector machine (SVM), decision tree, and random forest have been utilized for the selection of the embryo [22], classification of ovarian response [23] and embryo [24], and prediction of the embryo implantation outcome [25], etc. Regarding the prediction of the COS outcome, although there have been some clinical evaluations toward the impact features [23,24], most of them are univariate analyses conducted between a single feature (e.g., AMH or AFC) and the cursory classification outcome of the COS. Besides, the existing works mainly evaluated the importance of the impact features according to a simple correlation analysis (e.g., correlation coefficient and risk/odd ratio), which cannot scientifically and accurately evaluate the importance of the impact features.

This study aims to develop a new regression model for the prognostic prediction of oocyte number, considering the comprehensive impact features. Based on the proposed model, the impact features are quantificationally evaluated and compared at the same level. Eventually, tailored COS strategies can be provided based on the scientific prediction result from the proposed model.

## 2. Materials and Methods

**Figure 1:**
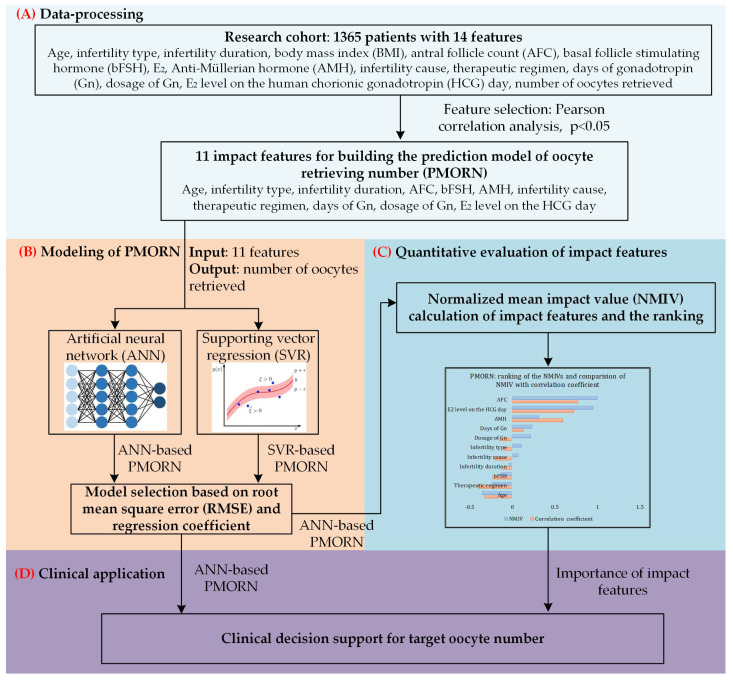
The overall framework of our study. (**A**) Data-processing toward the research cohort with 1365 sets of clinical data; Pearson correlation analysis is conducted to identify the impact features with significant correlation value. (**B**) Based on the two types of methods, ANN and supporting vector regression (SVR), two prediction models of the number of oocytes retrieved are built; the model selected is based on the training and prediction results. (**C**) Quantitative evaluation of the impact features, and ranking the importance of them. (**D**) Clinical application of the proposed model.

The overall framework of the proposed work is shown in Figure 1.

### 2.1. Data Retrieving and Processing

A total of 1365 women seeking the IVF treatment in the Renming Hospital of Wuhan University between October 2019 and December 2020 are taken as a research cohort for building and validating the proposed models, with the demographic and clinical properties listed in Table 1. The 14 potential impact features that have a close relationship with the COS process are considered when building the proposed models, which are the age, infertility type, infertility duration, BMI, AFC, bFSH, E_2_, AMH, infertility cause, therapeutic regimen, days of Gn, dosage of Gn, E_2_ level on the human chorionic gonadotropin (HCG) day, and the number of oocytes retrieved. The features are selected before building the proposed models, which is based on the univariate Pearson correlation analysis in the Real Statistics Resource Pack, with the significance (*p*-value) listed in Table 1. The correlation analysis is conducted between the number of oocytes retrieved (i.e., the COS outcome in this work) and the rest of the 13 impact features, and these with *p* < 0.05 are selected as impact features for building the proposed model.

### 2.2. Machine Learning-Based Modeling of the Number of Oocytes Retrieved

In this section, two popular machine learning methods for the non-linear regression problem are utilized to build the prediction model of oocytes retrieved and number (PMORN), which are the ANN and supporting vector regression (SVR). For the proposed PMORN, 11 features that have the *p* < 0.05 (as listed in Table 1) are selected, which are the age, infertility type, infertility duration, AFC, bFSH, AMH, infertility cause, therapeutic regimen, days of Gn, dosage of Gn, and the E_2_ level on the HCG day. These 11 features are taken as the input for building the PMORN while the number of oocytes retrieved is taken as the output.

#### 2.2.1. ANN-Based PMORN

The artificial neural network is a machine learning model inspired by the biological neural network of animals’ brains. The ANN has a wide variety of applications for classification and regression problems. By choosing the appropriate hyperparameters, the ANN can, in theory, approximate any type of nonlinear function; therefore, it is potentially a good model for building the PMORN.

**Figure 2:**
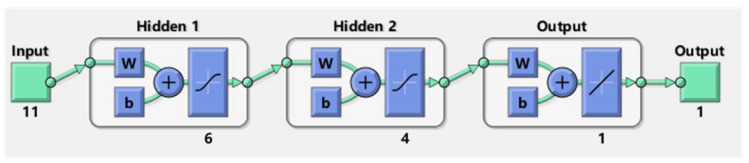
The structure of the proposed ANN.

In general, the proposed ANN is composed of three elements: the input layer, the hidden layers, and the output layer. The structure of the proposed ANN is shown in Figure 2. Regarding the input layer, it has 11 features, while the output layer has only 1 feature. The hyperparameters, such as the structure of hidden layers and the layer number are determined by a trial-and-error method. The proposed ANN has two hidden layers that have 4 and 6 neurons, respectively.

When training the ANN, 70% percent of the instances are utilized for training, while the rest is used for validation and testing of the trained network. In the training process, the algorithm of Levenberg-Marquardt is utilized to iteratively update the parameters of ANN, with the activation function of Sigmoid and learning rate of 0.01, L_2_ regularization is applied to avoid overfitting the model.

#### 2.2.2. SVR-Based PMORN

The SVR is also a popular regression algorithm of machine learning, which is similar to the SVM that is widely utilized in the classification problem. As an alternative to the ANN, the SVR-based PMORN is constructed in this study. For the proposed SVR, the input and output are the same as these of the ANN-based PMORN. When training the SVR, its hyperparameter is determined according to the “OptimizeHyperparameters” function of the SVR model: the gaussian kernel function is utilized, the width band epsilon is set as 0.5, the strategy of cross-validation is utilized with 10 folders to reduce the overfitting of the model, and the sequential minimal optimization is selected as the solver for training the SVR.

### 2.3. Quantitative Evaluation of the Impact Features

To quantificationally evaluate the effect of the impact features on the outcomes of the proposed models, a metric called the mean impact value (MIV) is calculated for each feature, which directly measures the importance of the feature toward the outcome. Assuming the $F\left(h_{1},h_{2},\ldots ,h_{n}\right)$ is a model constructed from the above-mentioned methods, where $h_{1},h_{2},\ldots ,h_{n}$ are the input features of the model and n=11 is the total number of features, the MIV for the *i*-th feature is calculated as

(1) $$miv_{i}=\frac{\sum_{1}^{m}\left[F\left(h_{1},\ldots ,1.1h_{i},\ldots ,h_{n}\right)-F\left(h_{1},\ldots ,0.9h_{i},\ldots ,h_{n}\right)\right]}{m}$$

where the m is the total number of women in the research cohort.

Given the model F, the MIVs of all features, i.e., MIV = ($miv_{1},miv_{2},\ldots ,miv_{n}$), can be calculated from Equation (1). To make the MIV of different features and models and compare at the same level, the MIV is normalized and represented as the normalized mean impact value (NMIV), and

NMIV = MIV/*max*(*abs*(MIV)) (2)

where in Equation (2), the *abs*(.) is the absolute value function; *max*(.) is the operation for achieving the maximal value of a vector.

Assume the NMIV calculated from Equation (2) is expressed as NMIV = $\left(miv_{1}^{*},miv_{2}^{*},\ldots ,miv_{n}^{*}\right)$, the *i*-th element $miv_{i}^{*}$ represents the importance of the corresponding feature, i.e., the degree the $h_{i}$ affects the outcome of the model (e.g., the number of oocytes retrieved in this study). A positive $miv_{i}^{*}$ means that increasing the value of $h_{i}$ could boost the predicted outcome, and the larger the $miv_{i}^{*}$ is, the more important $h_{i}$ could be for affecting the outcome, and vice versa.

The NMIV, as defined from Equations (1) and (2), can be utilized to directly and precisely quantify the effects of the input features on the outcome of proposed models. Based on the definition, the NMIV can be regarded as the normalized average partial derivative of the F toward its variables $h_{1},h_{2},\ldots ,h_{n}$ in calculus.

### 2.4. Statistical Platform

The software of MATLAB (version R2021a) is utilized to implement the machine learning algorithms and conduct the data analysis in this study.

## 3. Results

### 3.1. Performance of the Proposed Model

The training results of the proposed PMORN based on the ANN and SVR are evaluated by the index of root mean square error (RMSE), which is 2.63 and 3.70, respectively, and listed in Table 2. The RMSE of the ANN-based PMORN is smaller than that of the SVR-based PMORN, demonstrating that the former has better overall training accuracy than the latter.

**Figure 3:**
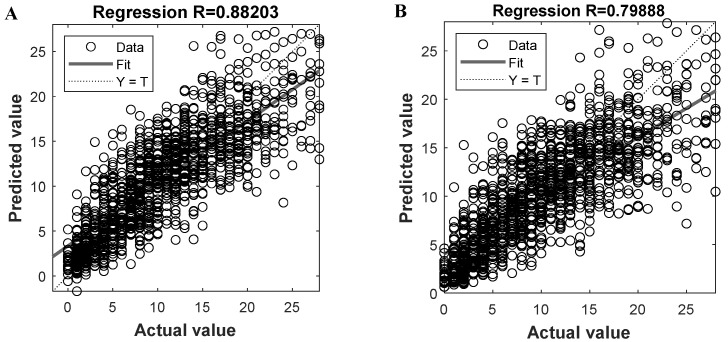
Prediction performance of the proposed models. (**A**) Regression coefficient R for the ANN-based PMORN; (**B**) regression coefficient R for the SVR-based PMORN. The horizontal and vertical axes, respectively, represent the actual value and the predicted value, which are denoted by the symbols Y and T, respectively.

Given the two trained models, we can get the predicted oocytes retrieving number and compare them to the actual value, with the results shown in Figure 3A,B, respectively. By plotting the predicted and actual results of all instances and fitting them into a line (i.e., the solid line in Figure 3), we know that the closer the line is to the diagonal Y = T (i.e., the dashed line), the better overall prediction performance the model could be. To evaluate the prediction performance, the regression coefficient R can be calculated for the fitted line; the larger the R is, the better the prediction result could be. For the proposed ANN-based PMORN and SVR-based PMORN, their R values are 0.882 and 0.799, respectively, as listed in Table 2, meaning that the ANN-based PMORN has better overall prediction accuracy than the SVR-based PMORN.

**Figure 4:**
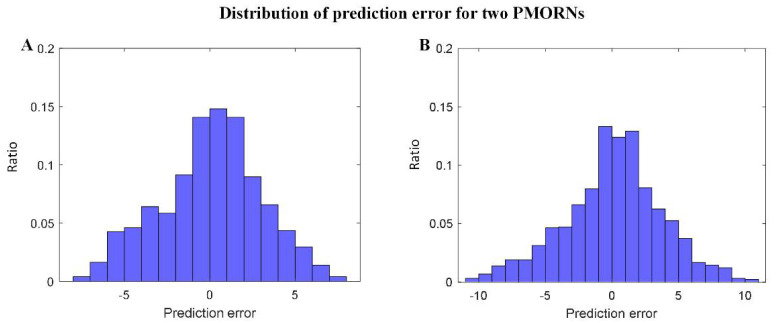
Distribution of the prediction error for the proposed models. (**A**) prediction error for ANN-based PMORN; (**B**) prediction error for SVR-based PMORN. The horizontal axis and vertical axis are the prediction error and the corresponding ratio for all the instances, respectively.

The statistical results regarding the prediction error of the two PMORNs are shown in Figure 4A,B, in which the distribution error generally obeys the rule of standard normal distribution. For the ANN-based PMORN, 66.91% of the instance has the prediction error ≤ 3, and 89.84% of the instance has the prediction error ≤ 5. Regarding the SVR-based PMORN, 60.87% and 81.71% of instances have the prediction error ≤ 3 and ≤5, respectively.

Based on the above results, we know that for the two PMORNs, both the training and prediction performance of ANN is much better than the SVR. Therefore, the ANN-based PMORN is selected as the final model for predicting the number of oocytes retrieved.

### 3.2. Quantitative Evaluation of Impact Features

Given the ANN-based PMORN, the NMIV of each feature can be calculated based on Equations (1) and (2) to quantificationally evaluate the importance of the 11 selected features.

**Figure 5:**
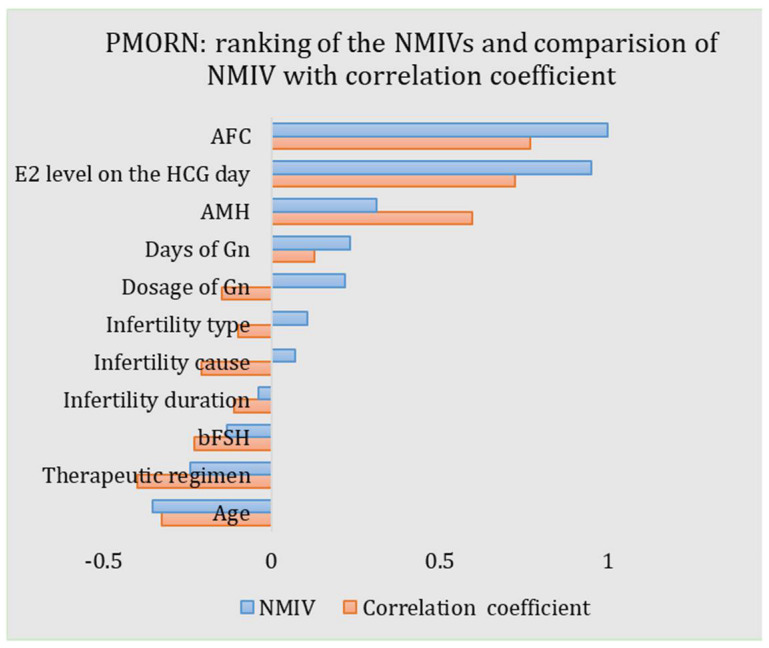
ANN-based PMORN: ranking of the features according to the NMIV, and the comparison of NMIV with correlation coefficient.

The NMIVs of all impact features for the PMORN are calculated and listed in Table 3 and shown in Figure 5. For an NMIV, its value quantificationally and directly measures the importance of the corresponding feature as compared to the other features; as the NMIV is normalized toward all features, its maximal value is 1. The sign of NMIV represents the positive or negative correlation between the feature and the outcome; if the sign is “+”, an increment of the value of the feature will boost the oocyte number and vice versa. For the PMORN which has 11 input features, the AFC has the largest magnitude of the NIMV (1.0), followed by the E_2_ level on the HCG day (0.951), the age (−0.354), the AMH (0.314), the therapeutic regimen (−0.241), the days of Gn (0.234), the dosage of Gn (0.219), the bFSH (−0.131), the infertility type (0.107), and the infertility cause (0.070), while the infertility duration has the least NIMV (−0.039).

The results listed in Table 3 demonstrate that the AFC has the highest priority affecting the number of oocytes retrieved, while the infertility duration is the least important feature. Results also demonstrate that, with the increase of AFC, AMH, days of Gn, Dosage of Gn, and E_2_ level on the HCG day, the general number of oocytes retrieved would also increase; the increase of age, infertility duration, and bFSH would, however, reduce the number of oocytes retrieved in return. For the other three impact features, i.e., infertility type, infertility cause, and the therapeutic regimen, as they are discretized features, their signs have no definite meaning.

For the proposed PMORN, when changing the settings (e.g., the architecture and hyperparameter) of its ANN, the importance of the impact feature, as measured by the NMIV, could indeed have some change, as it is calculated based on the PMORN. However, as our model is the one that has the best modeling performance, the NMIV, as calculated for the impact features (the AMH, AFC, bFSH, etc.), is also the most convincing result.

## 4. Discussion

In this study, a regression model for predicting the COS outcome is constructed based on two types of machine learning methods of ANN and SVR. The proposed models can yield good results in both training and prediction processes, and the feasibility that the number of oocytes retrieved is modeled via powerful machine learning methods as well as the sufficient retrospective clinical data is validated. Besides, the proposed regression models enable the quantitative evaluation toward the impact features of the two models, which provides the accurate ranking and evaluation for these features. To our best knowledge, we are the first to present a regression model for the COS that has good prediction results on the number of oocytes retrieved; also, there has never been any approach that can precisely evaluate the importance of comprehensive features, such as these conducted in our work.

Regarding the two types of machine learning methods, i.e., the ANN and SVR, the ANN is far superior to the SVR in terms of both the training accuracy (as measured by the RMSE, 2.63 vs. 3.70) and the prediction error (as measured by the regression coefficients R, 0.882 vs. 0.799). This is because the models to be constructed essentially have a high degree of nonlinearity and the input of the model contains both continuous features (e.g., AFC, AMH, and bFSH) and discretized features (e.g., infertility type and infertility cause). As the ANN can theoretically approximate any type of nonlinear model and can handle different types of input features, by carefully choosing the hyperparameters of the network, the ANN-based model is much better than that from the SVR. Therefore, the ANN-based PMORN is taken as the final model for predicting the COS outcome.

When constructing the PMORN, it has three major challenges: (1) three out of a total 11 features are discretized that have multiple conditions (i.e., the infertility type, infertility cause, and therapeutic regimen, as listed in Table 1); (2) the PMORN is a regression model constructed for the precise estimation of the oocyte number; and (3) the ovarian response mechanism of women is very complex, making it essentially difficult to be represented by an ideal mathematical model. These three issues make the construction of the PMORN with very good performance (high r-value) a challenging problem. Regarding the prediction accuracy, the regression coefficient of our model is 0.882, with 89.84% of the women having the prediction number ≤ 5. Although it is far from ideal, its performance is as good as the state-of-the-art work in [21,26], in which only cursory classification models on the COS outcome were built; they are much simpler than our method. Regarding the prediction accuracy, the area under the curve (AUC) (which is an index to reflect the classification accuracy, similar to our regression coefficient) is 0.859~0.903 and 0.892~0.897 for [21,26], respectively. As building the regression model is internally much more challenging, the prediction performance of the proposed PMORN is at least at the same level as [21,26], if no better.

As the proposed ANN-based PMORN is a regression model with comprehensive features, they enable us to conduct the precise evaluation of the features, as measured by the NMIV defined in Equation (2). Different from the univariate correlation coefficients (i.e., the r-value obtained from the Pearson correlation analysis and listed in Table 3), which is a coarse measure of how strong the relationship is between the feature and the outcome, the NMIV is an accurate and comprehensive reflection on how much the change of a feature (e.g., increase or decrease for 10%) can affect the COS outcome. For the NMIVs as listed in Table 3, they are compared with the corresponding Pearson correlation coefficients, with the results shown in Figure 5. Based on the comparison results, we have three findings: (1) The NMIV, in general, has a similar trend as the correlation coefficients. (2) The AFC has the largest NMIV (1.0) and the largest correlation coefficient (0.768) for the PMORN, followed by the E_2_ level on the HCG day (NIMV and r-value is 0.951 and 0.723, respectively). (3) For continuous impact features, the NMIV in general has the same sign as the correlation coefficient (AFC: 1 vs. 0.768; E_2_ level on the HCG day: 0.951 vs. 0.723; AMH: 0.314 vs. 0.596; days of Gn: 0.234 vs. 0.128; infertility duration: −0.039 vs. −0.11; bFSH: −0.131 vs. −0.23; and age: −0.354 vs. −0.325.). The only exception is the dosage of Gn, while its NMIV is a positive value of 0.219 but the correlation coefficient is −0.148; in general, more dosage of Gn will increase the number of oocytes retrieved in the IVF practice [25]; therefore, the negative correlation coefficient (−0.148) is not reasonable, which in return validate that the NMIV is a more accurate and reasonable index for ranking the importance of the COS outcome. Regarding the three discretized features (i.e., the therapeutic regimen, the infertility cause, and the infertility type), they are first encoded into some values and then sent for training and validation of the proposed model; the sign of their NIMVs has no definite meaning. Therefore, comparing the signs of their NMIVs with those of the correlation coefficients is meaningless.

For the proposed PMORN, the feature of AFC (NMIV = 1.0), E_2_ level on the HCG day (NMIV = 0.951), age (NMIV = −0.354), and AMH (NMIV = 0.314) have significant NMIV, meaning that they are the dominant features affecting the ovarian response, which is in accordance with the findings in [13,27]. The AFC has the most significant NMIV as compared to the other features, which matches with the findings in the early work [12,28,29,30] that (1) the AFC is highly correlated with the number of recruited follicles and the retrieved oocytes, and (2) the AFC is regarded as the best univariate predictor of ovarian reserve and determines the number of follicles that grows in response to Gn stimulation [13]. Besides, our work reveals that the E_2_ level on the HCG day (NMIV = 0.951) ranks in second place among all impact features, which matches with the finding that the number of oocytes retrieved increases with the amount of E_2_ level on the HCG day [29], as this feature somehow measures the response of the ovary to the COS and thus indirectly reflects the quality and quantity of the oocyte [31]. What is more, the NMIV of age is −0.354, meaning that age has a significant effect on the oocyte retrieving No, and the older the woman is, the more difficult it is to retrieve more oocytes in general [10]. Regarding the AMH, although recent studies claimed it has the highest importance for the ovarian response [32,33], our work confirmed its importance, and the larger the AMH is, the more oocytes it could retrieve in general; however, the status of AMH is weaker as compared to the AFC, E_2_ level on the HCG day, and the age. Results in our work suggest that we might need to re-evaluate the priority of the AMH and AFC on affecting the number of oocytes retrieved. In addition, we also find that the therapeutic regimen, days of Gn, dosage of Gn, bFSH, and infertility type have obvious influence on the ovarian response, as revealed in [27,34]. Regarding the infertility duration and infertility cause, their NMIV value is very small (−0.039 and 0.07), meaning they have very little effect on the COS outcome.

The proposed PMORN is constructed by mining the knowledge from the extensive retrospective clinical data via the machine learning method and therefore embedding the experiences of many experts. The proposed model has three potential applications. Firstly, scientific prediction on the outcome of the COS (i.e., the number of oocytes retrieved) can be achieved, based on which the potential IVF risks can be identified in advance, e.g., the predicted number of oocytes retrieved is too low (poor ovarian response with number <6—risk of IVF cycle being canceled) or too high (high ovarian response with number >18—suffer from potential OHSS) [35]. Besides, the proposed PMORN has good potential for clinical application. For a woman (seeking IVF treatment) with the given biomarkers, such as the age, infertility type, infertility duration, AFC, bFSH, AMH, and infertility cause, the proposed PMORN can be used to predict the oocyte number for tailoring the COS strategies, such as the therapeutic regimen and dosage of Gn. To achieve the target oocyte number (e.g., 16 oocytes), we could iteratively adjust the COS strategies according to the predicted results from the PMORN and the NMIVs of the COS features. For example, when given a certain dose of Gn, if the predicted oocyte number is less than the target value, we could increase the dose of Gn, as its NMIV listed in Table 3 is a positive value and vice versa. The dose of Gn is iteratively adjusted according to the trial-and-error predicted oocyte number; eventually, the dose of Gn is chosen as the value that yields the target oocyte number. Although the proposed PMORN has some prediction errors, the COS strategy tailored based on our model could serve as a credible reference to support the decision making in the COS process. Finally, the quantitative and precise evaluation of the impact features could help understand/explain the outcome of ovarian response and reveal the unknown mechanism behind it.

The proposed PMORN considers the comprehensive impact factors, including some special conditions, such as the PCOS ovulatory obstacle in the factors of infertility causes (refer to Table 1). Based on the study conducted in [14,36], we know that PCOS, especially the one with a high AMH level, could significantly affect the outcome of COS; a model (e.g., the nomogram [14]) that is suitable for tailoring the COS strategy in a general condition would, however, seem inadequate for PCOS women. For the proposed PMORN, it is a highly nonlinear model considering a certain number of PCOS cases (112 PCOS women); therefore, it could have the capability of handling the PCOS women. However, to investigate the effects of PCOS on the COS outcome more precisely, a COS outcome prediction model that is pertinent to PCOS women could be built based on the same method with enough clinical PCOS data. In this way, a potentially better model could be built for PCOS women.

Regarding our current work, it has three major limitations. First, the proposed model is constructed based on a single source of data collected in our reproductive medicine center; more data from the other medical organizations would further consolidate the findings of our work. In addition, the other issues in the ART process, such as the oocyte/embryo quality and IVF outcomes (pregnancy and live birth results), are not discussed in our current work. Finally, as listed in Table 1, the comprehensive multiple causes of infertility and heterogeneous treatment regimens are considered when constructing the PMORN. Considering only one or two types in these two features (e.g., the PCOS ovulatory obstacle) could, however, reduce the complexity of the modeling process and thus may improve the modeling performance. These three limitations will be addressed in our future work.

## 5. Conclusions

A regression model called PMORN is proposed that can accurately predict the number of oocytes retrieved. Based on the proposed model, the impact features are quantificationally evaluated: for the PMORN, the AFC and E_2_ level on the HCG day have the highest importance, followed by the age, AMH, therapeutic regimen, days of Gn, dosage of Gn, bFSH, infertility type, infertility cause, and infertility duration. The proposed models could serve as a scientific tool to predict the ovarian response and enable a customized, individual treatment of the COS for subsequent IVF cycles.

## Figures and Tables

**Table 1:** Demographics and clinical properties of the research cohort.

Features		Values	*p*-Value
Age		32.44 (21–50)	<0.001
Infertility type	Primary infertility	680 (49.82)	<0.001
	Secondary infertility	685 (50.18)	
Infertility duration		3.6 (0–22)	<0.001
BMI		22.27 (15.0–36.2)	0.594
AFC		19.60 (2–65)	<0.001
bFSH		9.37 (0.97–151.65)	<0.001
E_2_		83.96 (3.92–5086.19)	0.312
AMH		3.15 (0.1–23)	<0.001
Infertility cause	Pelvic and fallopian tube factors	444 (32.53)	<0.001
	Polycystic ovary syndrome (PCOS) ovulatory obstacle	112 (8.21)	
	Decreased ovarian reserve	173 (12.67)	
	Endometriosis and uterine factors	72 (5.27)	
	Multiple factors	494 (36.40)	
	Others	70 (5.13)	
Therapeutic regimen	Long protocol	222 (16.26)	<0.001
	Super-long protocol	385 (28.21)	
	Antagonist regimen	332 (24.32)	
	PPOS	309 (22.64)	
	Others	117 (8.57)	
Days of Gn		10.51(0–55)	<0.001
Dosage of Gn		2270.15 (0–6262.5)	<0.001
E_2_ level on the HCG day		2284.60 (57.41–19,432.60)	<0.001
Number of oocytes retrieved		11.18 (0–29)	/

Values are represented as the number of women (%) or average (range).

**Table 2:** RMSE and regression coefficient of the two PMORNs.

Model	ANN-Based PMORN	SVR-Based PMORN
RMSE value	2.63	3.70
Regression coefficient	0.882	0.799

**Table 3:** NMIVs and correlation coefficients of the impact features.

Features	Age	Infertility Type	Infertility Duration	AFC	bFSH	AMH	Infertility Cause	Therapeutic Regimen	Days of Gn	Dosage of Gn	E_2_ Level on the HCG Day
NIMV	−0.354	0.107	−0.039	1.0	−0.131	0.314	0.070	−0.241	0.234	0.219	0.951
r-value	−0.325	−0.1	−0.11	0.768	−0.23	0.596	−0.209	−0.398	0.128	−0.148	0.723

## Data Availability

Data in this study can be provided upon request.
